# NEP+: A Human-Centered Framework for Inclusive Human-Machine Interaction Development

**DOI:** 10.3390/s23229136

**Published:** 2023-11-12

**Authors:** Enrique Coronado, Natsuki Yamanobe, Gentiane Venture

**Affiliations:** 1 Industrial Cyber-Physical Systems Research Center, National Institute of Advanced Industrial Science and Technology (AIST), Tokyo 135-0064, Japan; n-yamanobe@aist.go.jp (N.Y.); venture@cc.tuat.ac.jp (G.V.); 2 Graduate School of Engineering, University of Tokyo, Tokyo 113-8656, Japan

**Keywords:** human-machine interaction, human-robot interaction, Industry 5.0, internet of robotic things, robotics middleware, social robotic

## Abstract

This article presents the Network Empower and Prototyping Platform (NEP+), a flexible framework purposefully crafted to simplify the process of interactive application development, catering to both technical and non-technical users. The name "NEP+" encapsulates the platform’s dual mission: to empower the network-related capabilities of ZeroMQ and to provide software tools and interfaces for prototyping and integration. NEP+ accomplishes this through a comprehensive quality model and an integrated software ecosystem encompassing middleware, user-friendly graphical interfaces, a command-line tool, and an accessible end-user programming interface. This article primarily focuses on presenting the proposed quality model and software architecture, illustrating how they can empower developers to craft cross-platform, accessible, and user-friendly interfaces for various applications, with a particular emphasis on robotics and the Internet of Things (IoT). Additionally, we provide practical insights into the applicability of NEP+ by briefly presenting real-world user cases where human-centered projects have successfully utilized NEP+ to develop robotics systems. To further emphasize the suitability of NEP+ tools and interfaces for developer use, we conduct a pilot study that delves into usability and workload assessment. The outcomes of this study highlight the user-friendly features of NEP+ tools, along with their ease of adoption and cross-platform capabilities. The novelty of NEP+ fundamentally lies in its holistic approach, acting as a bridge across diverse user groups, fostering inclusivity, and promoting collaboration.

## 1. Introduction

The rise of machine-centered approaches has led to innovations, sometimes neglecting human considerations [1]. Emerging paradigms like Industry 5.0 [2], and Society 5.0 [3] advocate a shift towards human-centric progress. The objective is to empower individuals, bridge knowledge gaps, enhance technology acceptance, and foster innovation across boundaries. In this context, our article addresses the challenge of deploying human-machine interactive applications in Industry 5.0 and Society 5.0. This challenge is transdisciplinary, involving diverse stakeholders from various fields and emphasizing accessible and holistic approaches [1].

With the increasing accessibility of computers to the general public, there has been a transition towards designing digital information systems for a more diverse user base. This transformation underscores a heightened focus on accessibility and user experience [4]. However, there is still a common misconception among expert developers that all users possess a high level of technological literacy. This can lead to creating complex tools and interfaces that are difficult for many people to use. Moreover, many advanced tools use complex jargon, posing challenges for even skilled users. Linux experts prefer such tools for control and precision, but they can be arcane, particularly command-line interfaces. This complexity hinders their use in applications for a broader, non-technical audience, especially non-technical stakeholders [5]. Traditionally, fields like robotics and the Internet of Things (IoT) have favored export-oriented approaches. Robotics frameworks are often tailored for Linux-based platforms and expert users, making them less accessible to non-technical individuals. While various robotics frameworks exist, few prioritize user experience and accessibility. Relevant export-oriented robotics frameworks are TalKRoBots [6], ROS [7], ROS 2 [8], E2M [9], Zoro [10], PDRA [11], YARP [12], and the RT-Middleware [13]. In contrast, recent developments in IoT have introduced user-friendly tools like Node-RED [14], designed to simplify IoT application integration and democratize technology for a broader audience. Similar tools under the end-user development [15] area are comprehensively discussed in [16,17] for IoT and robotics. These tools are part of an emergent transformation that seeks to democratize technology by enhancing the accessibility of emergent technologies for a wider audience.

To foster the democratization of technology, this article introduces the preliminary version of NEP+ (Network Empower and Prototyping Platform). We designed NEP+ as a comprehensive framework, seamlessly integrating a quality model and a software platform purposefully designed to facilitate the development and design of interactive applications involving both physical and digital agents. We aimed to make NEP+ accessible to a wide-ranging audience, from novices to experts. To achieve this objective, we present two pivotal contributions. Firstly, the NEP+ quality model lays down a set of guiding principles for the creation of interactive systems, placing a strong emphasis on prioritizing user experience (UX) and empowering potential users and co-designers of human-machine interactive applications. Secondly, the NEP+ software platform builds upon the communication libraries presented in [18,19]. What distinguishes NEP+ from these previous works is the introduction of a platform encompassing a middleware layer, a command line interface, and a comprehensive suite of user-friendly interfaces, providing developers with the tools and resources needed to harness these libraries to create modular, complex, and distributed applications.

It’s important to highlight that this article primarily focuses on presenting the functional features and implementation architecture of NEP+. Consequently, evaluating NEP+ user interfaces with novice users is not within the immediate scope of this work. Nonetheless, this article incorporates vital pilot tests. These initial tests aim to identify potential usability issues and gather valuable feedback. This feedback is instrumental in refining the user interfaces for subsequent and more comprehensive experiments with broader users.

## 2. Contributions and Key Distinctions from Similar Solutions

NEP+ represents a significant departure from conventional frameworks like ROS and ROS 2. Like in the case of Node-RED, NEP+ graphical user interfaces leverage Node.js, a JavaScript runtime compatible with various operating systems, to provide support for Windows, macOS, and different Linux users. Nevertheless, where Node-RED is bound to Node.js and flow-based programming paradigms, NEP+ provides a more versatile developer environment that accommodates multiple programming languages and can be adapted to traditional and visual programming styles. The most recent version of NEP+ encompasses three essential elements: (i) a holistic and human-centered quality model that guides software and application development, (ii) a middleware layer that facilitates the seamless integration of software modules, and (iii) a set of interfaces that simplify the utilization of sensing, human perception, and behavior orchestration algorithms with minimal effort and experience required. Contributions of this article are presented and organized as follows:

- Section 3 introduces the NEP+ development model.
- Section 4 provides an in-depth overview of the middleware layer and interfaces offered by NEP+.
- Section 5 briefly describes ongoing research projects employing NEP+.
- Section 6 describes technical features of NEP+ and its comparison with state-of-the-art robotics frameworks.
- Section 7 reports a usability evaluation of the NEP+ interfaces and compares the effort required to construct a basic application using NEP+ versus a modern state-of-the-art robotics framework.

## 3. NEP+ Human-Centered Quality Model for Application Development

In this section, we introduce a quality model, defined in [20], which describes quality aspects of software modules and applications designed for interactive systems from a human-centered perspective. We start by clarifying the general meaning of human-centered. Then, we present and explain the elements composing the proposed model.

### 3.1. What Is Human-Centered

Human-centered is a complex and multidimensional concept with overlapped definitions that produce disagreements in the scientific community [1]. Cooley states in [21] that the central assertion of human centredness is that "people must be always put before machines, however complex or elegant that machine might be". This requires a change in the design philosophy from the traditional focus on increasing machine performance and functionalities (an approach often omitting human factors) to new approaches focused on the provision of tools supporting human skills, limitations, needs, and ingenuity [21]. Therefore, human-centered technologies must be able to empower humans rather than replace them with machines. In this context, Industry 5.0 and Society 5.0 promote the use of technology to empower and promote the talents and diversity of humans [1]. This article proposes a quality model for developing interactive systems centered on humans, in line with the vision of human-centered technology design.

### 3.2. Importance of User Experience (UX) in Human-Centered Approaches

Human-centered design seeks to develop products and services that prioritize functionality, usability, accessibility, and user enjoyment [22]. The user experience (UX) is pivotal in achieving this objective, enabling designers to comprehend and cater to users’ needs, preferences, and constraints [1]. Popular UX general-purpose models that serve as guides for practitioners and researchers, and upon which research is built, include the UX honeycomb [23] and Hancock’s Hedonomic Pyramid [24]. Other very recent UX models for interactive systems have been introduced in [1,25] as an evolution of Hancock’s Hedonomic Pyramid.

### 3.3. Proposed Human-Centered Model

**Figure 1:**
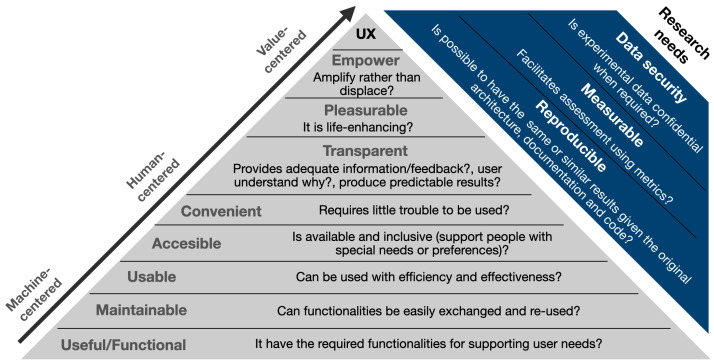
Proposed model of needs for interactive applications. The presented version of NEP+ focuses on the User Experience (UX) pyramid of needs. The support of research needs is left for future iterations/publications.

This article formulates a holistic model for developing software for interactive systems, summarized in Figure 1. Elements of this diagram represent the design goals or road-map of the development of NEP+ and the modules developed with this framework. These design goals are presented in a pyramid diagram, similar to Maslow’s Hierarchy of Needs [26] and adapting some of the UX elements available in the UX honeycomb and [27]. At the pyramid’s base are the machine-centered elements composing the basic functionalities for enabling machines (e.g., robots) to achieve aimed tasks and re-use functionalities. Human-centered factors aim to reduce the cognitive load that represents building interactive applications. Finally, value-centered elements aim to greater user satisfaction by providing meaningful and enjoyable interactions with graphical user interfaces. Some additional user and research needs are described outside the pyramid of needs, which can be presented in practical and academic scenarios. However, fulfilling these additional needs is outside the scope of this article. Elements of the proposed model are described below.

#### 3.3.1. Useful/Functional

Usefulness is an essential feature of any human-machine interaction and software system. For this, it is required to provide a basic set of core functionalities that, after integration, enable users to achieve their goals. In advanced applications, software architectures are built by integrating several building blocks. Each building block or node can embed one or more functionality. In this direction, ISO/IEC 25010 [27] defines functional suitability for software systems as "the degree to which a product or system provides functions that meet stated and implied needs when used under specified conditions".

#### 3.3.2. Maintainable

ISO/IEC 25010:2011 [27] defines maintainability as a characteristic that "represents the degree of effectiveness and efficiency with which a product or system can be modified to improve it, correct it or adapt it to changes in the environment, and in requirements". A relevant approach for producing maintainable systems is the development of modular (i.e., discrete and isolated) software components. In robotics, these components are often identified as nodes. Ideally, these nodes must provide a correct abstraction level, enabling their easy re-use in other systems (i.e., re-usability) and enabling users to produce high-quality interactive applications with less effort [17,28]. If this abstraction is too high, it will provide less control to developers. If this abstraction is too low, it will increase the expertise and effort of novice programmers required to understand, build, modify, and execute applications [28].

#### 3.3.3. Usable

According to ISO 9241-11:2018 [29], usable systems are those capable of achieving a task with effectiveness, efficiency, and satisfaction. Despite this standardized definition, the Human-Computer Interaction (HCI) community lacks a consensus on the exact definition of usability [30]. In general, usability encompasses creating interactive systems that are effective, efficient, engaging, error-tolerant, and easy to use [1].

#### 3.3.4. Accessible

ISO 9241-11:2018 [29] defines accessibility as "the extent to which products, systems, services, environments, and facilities can be used by people from a population with the widest range of user needs, characteristics and capabilities to achieve identified goals in identified contexts of use". We can differentiate two types of meaning for accessibility in software systems as inclusive and available. On the one hand, inclusive software often refers to ensuring that places, products, and services are fully open to and usable by people with special needs or different types of skills and preferences. On the other hand, accessible software can also refer to available artifacts that users can download and use for free at any time or on their preferred device and can be adapted to outside-laboratory contexts. For the purpose of this article, we adopt the latter definition.

#### 3.3.5. Convenient

Oxford dictionary defines convenient as "useful, easy or quick to do; not causing problems." Therefore, the software that is convenient maximizes comfort, involves little trouble, and is easy to get started (i.e., reducing training or learning). In this context, we consider compatibility and portability as crucial elements for reducing troubles for users, enabling them to use their preferred devices or current resources to execute or design interactive applications. On the one hand, ISO/IEC 25010 [27] defines compatibility as the "degree to which a product, system or component can exchange information with other products, systems or components, and/or perform its required functions while sharing the same hardware or software environment". On the other hand, ISO/IEC 25010 [27] defined portability as the "degree of effectiveness and efficiency with which a system, product or component can be transferred from one hardware, software or other operational or usage environment to another". Ideally, this feature requires developing software that adapts or evolves to different operational or usage environments (i.e., adaptability) and provides an easy, lightweight, and efficient installation and/or uninstallation (installability).

#### 3.3.6. Transparent

Transparency is a relevant feature for enhancing users’ trust in interactive systems [31,32]. Transparency can be used as an umbrella for other overlapped concepts, such as human-readable (i.e., display information that humans can naturally read and analyze), visibility (informed users about the status of the system through appropriate feedback), and explainability (i.e., users can understand why and how machines produce a result) [1]. Therefore, software artifacts must produce predictable results and provide enough information to enable users to be aware of the state of the system, recognize and recover from errors, and monitor the interaction between robotics building blocks.

#### 3.3.7. Pleasurable

Cognitive science studies suggest that humans emotionally connect the performance of some interactive products with their visual appeal [33]. This phenomenon is the aesthetic-usability effect [34] and suggests that providing relevant, life-enhancing, and valuable user experiences must also consider emotional and user interface (UI) design aspects. However, aesthetics must support functionalities instead of making usability issues more tolerant [34]. This argument is also supported by Helander et al. [35], which describes that "in emotional design, pleasure and usability should go hand in hand, as well as aesthetics, attractiveness, and beauty". Therefore, the main challenge is to create aesthetically pleasing interfaces that, at the same time, are intuitive, consistent, and coherent.

#### 3.3.8. Empower Rather than Replace

Labor is a fundamental and universal dignity aspect of humans regardless of social, political, or economic contexts [36]. As described in [37], one of the main characteristics of digitalization is its disruptive quality in areas such as the labor market. An alternative for "disrupting disruption" of AI systems is to empower humans, such as is proposed with the concept of digi-grasping [37], end-user development [38], human-robot collaboration [1] and participatory design [39]. We believe that the design of emergent technologies must put humans at the center. Therefore, humans must be able to select or personalize which type of software, hardware, or AI model better satisfies their interests and needs (e.g., fully- or semi-autonomous, multi- or single-purpose, black-box or white-box, complex or simple). Moreover, more advanced systems can learn from interacting with humans to improve job satisfaction and performance (e.g., in a manufacturing environment) or to engage people for more time (e.g., in a social setting).

#### 3.3.9. Research Needs

In academic contexts, reproducibility is fundamental. For this, systems using the same architecture, the same documentation for installation and configuration, and the same code must obtain the same or similar results. This process requires collecting and analyzing human data using objective or subjective methods. Therefore, research systems will need tools to assess and validate studies based on metrics [1]. Portable code and architectures that junior students can use and a common evaluation framework are desired for facilitating research activities. Finally, systems collecting potentially sensitive data from humans must provide solutions to ensure that data obtained from human experiments remains confidential [1].

### 3.4. Satisfying Proposed Human-Centered Model Using NEP+ Software Platform

Table 1 provides an overview of how NEP+ addresses the dimensions discussed in Section 3.3 and maps these dimensions to the corresponding sections of this article. Many specific quality elements are primarily addressed through the provision of a robust software architecture and accompanying tools, ensuring features such as usefulness and maintainability. These aspects are facilitated by the NEP+ middleware layer, as described in various sections of this article. Furthermore, value-centered dimensions can be assessed by examining the impact and outcomes of the tools on the community. This is exemplified in Section 5, where we demonstrate the real-world impact of NEP+ in the context of social robotics. However, certain human-centered dimensions require an iterative interaction design process for effective realization. This iterative process encompasses ideation, prototyping, and testing stages. In this context, this article represents an initial effort toward addressing the proposed quality elements, setting the stage for further exploration and refinement in subsequent work.

## 4. NEP+ Middleware Layer and Interfaces

**Figure 2:**
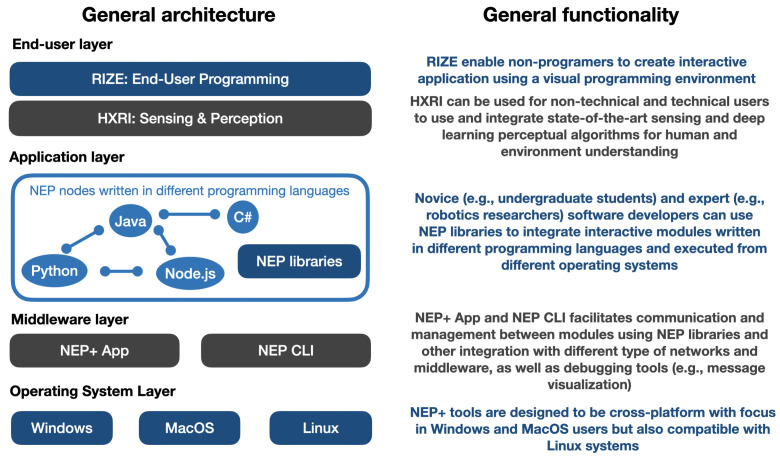
General overview of NEP+ tools and description of their main functionalities.

Figure 2 presents a general overview of the current NEP+ tools, comprising three layers: middleware, application, and end-user. The middleware layer facilitates the integration of distributed systems using ZeroMQ sockets. The application layer provides a set of libraries that developers can use to connect modules written in different programming languages. The end-user layer consists of all user interfaces developed using the NEP+ software architecture, enabling non-programmers to prototype or execute applications. This section describes the middleware capabilities and interfaces that compose NEP+.

### 4.1. NEP+ App

**Figure 3:**
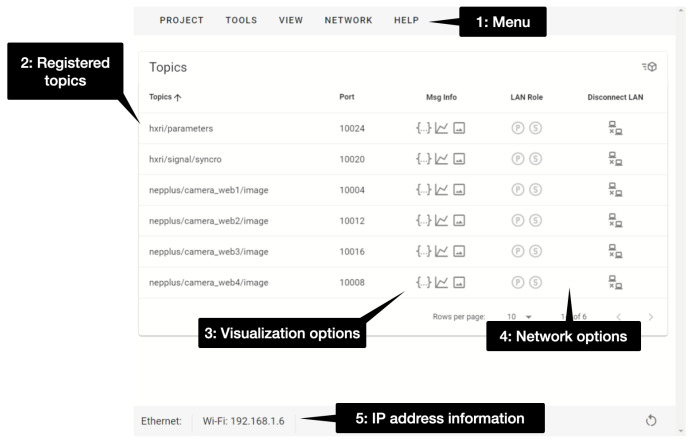
NEP+ App interface version 0.0.4. The main elements of this interface are: (1) a main menu allowing users to select various interface tool, (2) a list of registered topics in the NEP+ network, (3) a set of visualization options for each topic, (4) additional network configuration choices specific to each topic and (5) essential information regarding the computer’s IP address.

The core of NEP+ is built upon ZeroMQ [40], an open-source, lightweight, and high-performance messaging library that provides a socket application programming interface (API) and supports advanced messaging patterns. ZeroMQ utilizes the ZeroMQ Message Transport Protocol (ZMTP) and is released under the GNU General Public License. One of its key strengths is its portability and universality, enabling seamless socket-based communication between software modules across nearly all modern programming languages and operating systems. ZeroMQ’s capabilities make it an ideal choice for constructing robotics and IoT modules that can seamlessly run on various devices and programming languages with minimal complexity. These attributes align well with the requirements outlined in our quality model. NEP+ libraries and tools provide a level of abstraction that simplifies the utilization of ZeroMQ, enabling straightforward development of interactive applications. At the heart of the NEP+ framework lies the NEP+ App, serving as the central interface responsible for managing distributed system connections through ZeroMQ sockets. Figure 3 shows the main menu of NEP+ App in its version 0.0.4, which was used in the experimental section. Details of how to use the NEP+ App 0.0.4 are available online [41]. The most relevant functionalities of NEP+ App are service discovery and message monitoring.

#### 4.1.1. Service Discovery

Service discovery plays a critical role in enabling the automated detection, registration, and management of services within distributed systems. This feature, unavailable in ZeroMQ, is essential for developing complex and heterogeneous software architectures. In the realm of robotics, services are often referred to as nodes, each necessitating a unique identifier. Nodes can encompass one or more channels or ports, serving as conduits for communication with other nodes within the software architecture. These channels’ functionalities are determined within each node’s code, designating them as either publishers (input channels) or subscribers (output channels). Each channel is further identified by a topic name. To enlist a publisher or subscriber within a distributed application, the corresponding node must transmit a connection request to the NEP+ App in JSON format.

#### 4.1.2. Message Monitoring

**Figure 4:**
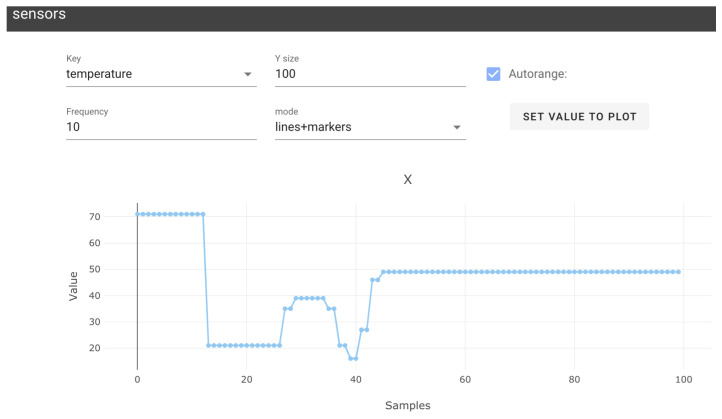
Example of message monitor using 2D plost using NEP+ App 0.0.4. Blue dots display data obtained in some specific topic over time. Blue dots visualize data acquired from a particular topic over time.

Messages sent between nodes can be visualized in separate windows. Depending on the message type, they can be visualized in a hierarchically organized text format, as 2D plots (Figure 4) or images.

### 4.2. NEP Libraries

Originally described in [18] and extended for supporting MATLAB in [19], NEP libraries abstract low-level sockets implementations for enabling inter-process communication functionalities in modern programming languages. For this initial version of NEP+, we have chosen ZeroMQ as the primary back-end framework over other message libraries, such as gRPC. However, we plan to provide solutions enabling the use and easy integration of more message libraries, such as socket.io and MQTT, gRPC, and nanomsg. ZeroMQ’s lightweight, low-latency messaging is ideal for our high-performance applications, specifically sensor networks. Its flexible communication patterns, including publisher-subscriber, meet several messaging requirements, including robotics applications. Unlike other potentially suitable alternatives, such as gRPC, ZeroMQ’s simplicity and transparency minimize the need for frequent message debugging. Furthermore, ZeroMQ’s publisher-subscriber pattern provides benefits such as separation of concerns, loose coupling, decentralized error handling, and message filtering, reducing the debugging effort. Additionally, we selected to build NEP+ on top of ZeroMQ due that do not require third-party software or complex installation steps and is compatible with almost any modern programming language and operating system. This includes Windows 11 and macOS Ventura, which ROS does not currently support. These features make ZeroMQ sockets a suitable solution for end-user software development. Some works, such as [42,43] have proved the technological suitability of ZeroMQ sockets. Furthermore, the literature reports some effort comparing the communication performance of ZeroMQ against similar solutions in [44,45]. These studies proved the suitability of ZeroMQ sockets in performance-sensitive applications. Kang et al. in [46] recently evaluated ZeroMQ against Message Queuing Telemetry Transport (MQTT) and OMG Data Distribution Service (DDS) for the deployment "of real-time applications and high-speed dissemination of massive data" in the Internet of Things. On the one hand, MQTT is defined in [47] as a publish/subscribe protocol for constrained devices requiring low-bandwidth and high-latency features under unreliable networks. On the other hand, DDS is an Object Management Group (OMG) standard, and middleware protocol designed to be used in critical domains requiring real-time and fine-tuned communication through quality of service (QoS) policies [48]. Results of [46] indicate that ZeroMQ has lower latency than DDS and MQTT for small (64B) and medium (2KB) messages. However, DDS latency outperforms ZeroMQ for large messages (32KB). We have also evaluated the communication performance of NEP libraries in [18].

### 4.3. NEP CLI

Node.js enables the creation of command-line tools that can be easily installed using the Node Package Manager (npm). We developed NEP CLI, a command line tool distributed as a package for npm, which provides an alternative solution for more traditional developers. This tool is platform-agnostic and easy-to-install. The only third-party dependency required to install NEP CLI is Node.js. After installing Node.js NEP CLI can be installed with the command npm install -g nep-cli. After this, the user can use execute nep commands in the preferred command line interface. A list of commands and a description of their functionality is available online [41]. The most relevant command is nep master. This command will provide the same service discovery capabilities as the NEP+ App. The main dependency of the NEP CLI package is the nep-js library. This JavaScript library provides the main middleware functionalities for both NEP+ App and NEP CLI. With nep-js, developers can create off-the-shelf software frameworks (i.e., ready-made software platforms that users can download, set up, and use) that embed core the middleware functionalities of NEP+ App and NEP CLI.

### 4.4. RIZE

The NEP+ interfaces are built upon the enhanced version of the Robot Interfaces from Zero Experience (RIZE) software architecture [38]. RIZE is specifically developed to empower non-programmers to create robot applications. RIZE comprises various components, including a visual programming environment, action-making modules, a blackboard for information sharing, and a decision-making engine based on Behavior Trees [49]. These components collaborate to facilitate the creation of robot applications by individuals without programming experience, allowing them to utilize their domain expertise and preferences in designing robot applications. A comprehensive description of RIZE is beyond the scope of this article, and interested readers can refer to [38] for further details.

### 4.5. HXRI

In response to the feedback received in Section 7, this article introduces the initial version of the Human-X Real-time Interaction (HXRI) interface. HXRI aims to simplify the development and experimentation process of applications that require collecting data from humans using a unified, user-friendly interface. Built on NEP+, HXRI enables the seamless integration of sensing and perceptual algorithms, such as body, hand, face, and emotion recognition, with other NEP+ interfaces or modules. The current version of HXRI (0.0.0.1) primarily focuses on vision algorithms, utilizing libraries like MediaPipe, OpenCV, and OpenVINO. It also facilitates the collection and transmission of sensory information from smartwatches, tablets, or smartphones to the HXRI application through the NEP+ App. This enables real-time interaction and the incorporation of sensor data into the HXRI framework for further processing and application development

### 4.6. Creating and Connecting Low-Level Components Using NEP+ Tools

In addition to its human-centered approach, NEP+ provides the flexibility to develop applications using a more low-level and traditional approach. This allows users to leverage NEP+ interfaces for common interaction tasks, such as emotion recognition, while still having the capability to work with the intricacies of low-level development. NEP+ primarily employs the Publisher-Subscriber pattern for module communication, and users can manage and monitor the software architecture using either the NEP+ App or the NEP CLI command lines. Users can easily add or replace nodes in the software architecture by locating the script’s path containing the node’s code and specifying the required launching commands and arguments. This approach can offer benefits such as rapid prototyping, fast integration of software modules, minimal training and software installation, and portability and compatibility with the newest (e.g., Windows 11 and macOS Ventura) or some older (e.g., Windows 8) operating systems.

NEP+ is not intended to replace existing state-of-the-art middleware but rather extend the capabilities of current software architectures. An example is the combination of NEP+ tools and ROS to build a complex Human-Robot Interaction (HRI) system using both NEP+ tools (for sensing and perception) and ROS (for robot control), as proved in [50], which uses a very early version of NEP libraries to integrate modules developed in ROS and Windows. NEP+ App 0.0.4 for Linux can link communication channels (topics) with a ROS architecture to facilitate compatibility and reuse of available ROS components. Currently, NEP+ supports a subset of ROS standard messages, but future iterations will enhance compatibility with ROS and other robotics middleware.

### 4.7. Creating Cross-Platform and Native Components Using NEP+ and Electron

The traditional motivation of most academic code and projects focuses on the performance of proposed algorithms or systems. This focus often produces systems with high computational costs or complex engineering requirements. Tools such as ROS and Docker make robot prototyping faster for expert developers but not necessarily easier in more human-centered scenarios [51]. In this context, off-the-shelf (i.e., ready-to-use) components can be an alternative for facilitating human-centered research activities, such as participatory design [51]. In fact, off-the-shelf or practical software provides a set of advantages often neglected in academic code, such as installability, compatibility, and a suitable user interface that facilitates their use for a wider audience. In the following paragraphs, we explain how NEP+ tools can be convinced with web technologies to produce off-the-shelf components for robotics that can be easily installed and used, such as most everyday-life applications.

**Figure 5:**
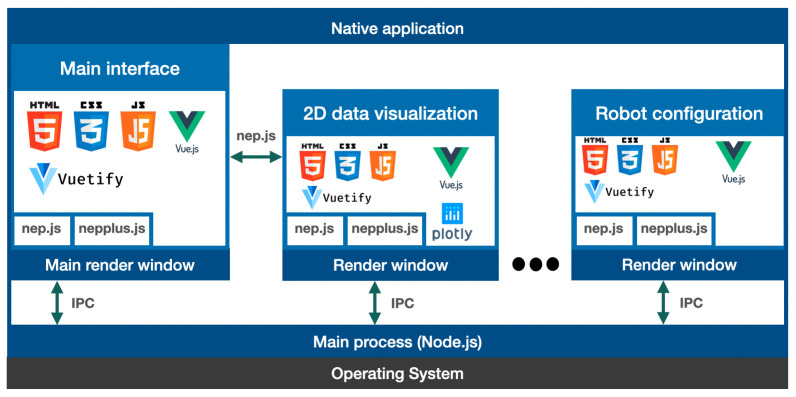
NEP+ App software architecture based in Electron and web-technologies. The main process manages a set of render windows (user interfaces) embedding robotics middleware functionalities (main interface) and other tools for helping in design robotics systems, such as data visualization and configuration of robot parameters (e.g., IP address).

Our proposed architecture for developing cross-platform and user-friendly components utilizes Electron [52], a framework that enables the creation of native applications for Windows, Linux, and OSX systems using web technologies like JavaScript, HTML, and CSS. By using Electron to generate native executables for NEP+ interfaces, users are not required to install additional third-party software. Figure 5 shows the software architecture of NEP+ App, which is mainly developed using Javascript frameworks. An Electron application comprises a single main process, which acts as the entry point and manages the application’s lifecycle, and one or more render processes that provide the user interface. Main and render windows communicate using an Electron feature denoted inter-process communication (IPC). NEP+ App comprises the main render process that provides most of the robotics middleware functionalities described in Section 4. This main render process can send messages using the IPC future of Electron to open or close other render windows when required by users. Each render window uses nep.js library to send messages between them. Each render window can use different Javascript frameworks, for example, 2D visualization of numerical data is performed using plotly.js [53]. We also developed the nepplus.js library to provide some helper functions when developing NEP+ interfaces, such as spawning non-node.js processes, such as a python script, setting the correct path of executables, saving and loading configuration files, and converting strings to JSON format, among others.

**Figure 6:**
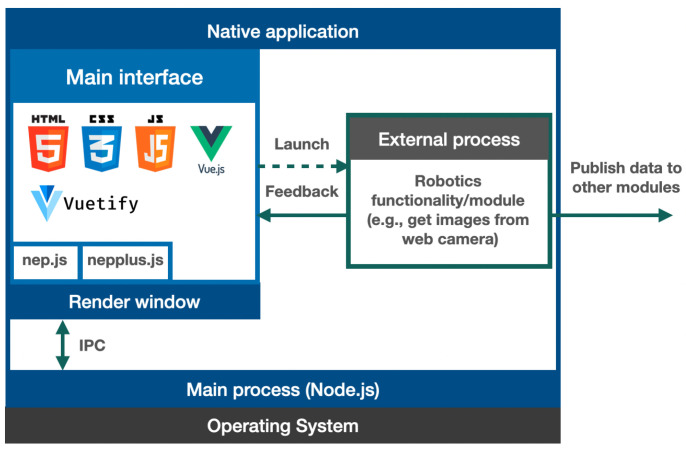
Basic software architecture of a NEP+ interface based in Electron. This off-the-shelf application embeds a robot functionality written in Python (external process).

Figure 6 shows the software architecture of basic NEP+ interfaces using Electron. In this architecture, the application comprises the main process, a render process, and an external process. The external process can be developed in Python and integrate a robotics functionality, such as getting images from a camera. The rendering process launches the Python script attaching the arguments selected by the user in the user interface. This script can be converted to an executable Windows program using tools such as [54]. This approach will enable the execution of scripts developed in Python without a Python version installed on the end-user computer. Furthermore, this script can send information about its state to the render window using the Python version of NEP libraries. The render window can use this information to suggest solutions to the user when an execution problem occurs. In addition, the executable can subscribe or publish data to other modules or NEP+ interfaces.

## 5. Example of Human-Centered Projects Supported by NEP+ Tools

Traditional robotics design methodologies are often machine-centered, primarily driven by technical considerations [1]. This approach typically involves teams of engineers who may not fully grasp the practical applications and real-world implications of the systems they create [55]. This leads to a disconnect from the needs and desires of end-users and the potential for addressing societal issues effectively through technology [56]. Human-centered methodologies provide an alternative to traditional approaches, emphasizing that users are the true experts in their lives [56]. These methods also acknowledge the value of domain experts in shaping design methodologies that result in better user experiences and address emerging technology adoption challenges [57]. Many human-centered methods promote the empowerment and inclusion of domain experts and users in co-design activities, involving them at various stages of development. This section introduces two real-world projects that utilize NEP+ to empower developers and domain experts in the design of robotics systems and platforms.

### 5.1. Yokobo, a Robot Powered by NEP Libraries

Yokobo is a non-anthropomorphic, non-vocal robot designed to enhance the connection between couples [58]. It was created through a user-centered and agile methodology inspired by the slow technology principle, earning recognition with the 2021 Kawaii Kansei Design Excellence Award [59] from the Japan Society for Kansei Engineering. The current software architecture involves two Raspberry Pi units, one for environmental sensing and the other for robot behavior control, with data exchange facilitated by NEP libraries [60]. The integration of Yokobo’s components was largely carried out by junior students in Japan with mechanical and electronics backgrounds but limited prior knowledge of software architectures for robotics. To address this mobility barrier, we developed a mobile application using NEP libraries to configure, monitor, and control Yokobo. This application, described in [61], is also used to facilitate the integration of Yokobo in a Smart Home.

### 5.2. Supporting Human-Centered Research Using RIZE

RIZE has played a pivotal role in bridging disciplinary and knowledge gaps across various Human-Robot Interaction (HRI) scenarios. It has facilitated collaboration among service users, social researchers, designers, and robotics engineers. The preliminary version of RIZE supported co-design activities using the Design Thinking (DT) methodology [62]. In this project, designers harnessed RIZE to create robot behaviors, contributing to a study on young retirees’ perceptions of robots in their homes [38]. RIZE has also been employed in co-design workshops using Participatory Design (PD) methodologies, actively engaging end-users in the conceptualization and design process of new technologies [39]. These workshops deepen users’ understanding of robotics technology and stimulate user-centered discussions. More insights into RIZE’s software architecture and its role in human-centered co-design activities can be found in [38].

## 6. Accessibility Comparison with Robotics Frameworks

This section compares NEP+ and similar frameworks from a human-centered point of view, specifically accessibility. Frameworks compared in this section have been selected based on the following criteria:

- They have a research and robotics focus
- They have been widely used or have future potential and emergence
- They are free and have an open-source license

Table 2 offers a comprehensive comparison of accessibility aspects between NEP+ and prominent robotics frameworks/middlewares, focusing on availability, interoperability, and inclusion. Availability is assessed by the number of supported programming languages, interoperability by the range of supported operating systems, and inclusion by the primary or typical user groups for each framework. According to the table, NEP+ and ROS2 support different programming languages, with NEP+ offering the potential for further language extensions, including Python 2 support, which is still an essential requirement for some robotics platforms like NAO/Pepper SDK. NEP+ also stands out in supporting a broader array of operating systems compared to ROS, YARP, and ROS2. According to [63], working with ROS frameworks implies some disadvantages. One of these disadvantages is that "ROS is mainly used in Linux systems, and it is not fully supported in other operating systems" [63]. While this issue can be trivial for many academic robotics researchers, it can become relevant for people working in other environments. An example is described by [63], which claims that "industrial manufacturers normally have their manufacturing execution system (MES) and the related infrastructures based on Windows". ROS2 and YARP are known to function on older macOS versions and new releases of ROS2 are often tailored to specific operating system versions. This can create inconveniences for users who need to update to the latest operating system versions to ensure security. NEP+ distinguishes itself by offering cross-platform, portable, and interoperable libraries, enabling the development of robotics systems on both older and modern operating systems. These advantages align with NEP+’s core objective of making robotics technology accessible to a diverse range of users with varying computational resources, backgrounds, and preferences.

## 7. Usability and Workload Evaluation of NEP+

In this section, we assess the usability of NEP+ tools to validate initial prototypes and gather impartial user feedback. Additionally, we measure the cognitive workload experienced by developers using NEP+. Due to COVID-19-related constraints and limited participant availability, we conducted a pilot test involving five participants from diverse nationalities. This approach aligns with the HCI community’s consensus that usability insights are often most effectively obtained from testing no more than five users [64].

The majority of participants in this pilot study reported having over five years of experience in programming software modules for robotics systems, with one exception being participant P4, who indicated having at least three years of experience. Among the participants, three individuals claimed to possess extensive experience using ROS, while the remaining two participants (P1 and P5) stated that they had a basic understanding of ROS. Since this pilot test was conducted within our institution and department, and the scope of this article does not involve a comprehensive or full-scale study, obtaining informed consent was not required, following our institution’s guidelines. The pre-questionnaire revealed an interesting finding: while all participants are ROS users, which is commonly associated with Linux/Ubuntu machines, most of them primarily utilize macOS and Windows for their research, work, and study activities. Additionally, regarding general activities, macOS is the preferred operating system among the participants. This highlights the diverse computational preferences of the participants, demonstrating the relevance and importance of providing cross-platform support. Therefore, participants of this experimental session can select to perform the tasks composing the proposed activities on either Windows or macOS. In most cases, participants used their computers for work (i.e., programming, writing papers, working from home, and performing administrative tasks) to complete the experiments. Otherwise, we provided them with a laptop with a clean installation of Windows 10 (compatible with ROS 2).

The research questions guiding this pilot study are:

- RQ1: Which is the perceived usability and feelings towards the current version of NEP+ interfaces?
- RQ2: Can developers create a basic robotic application from scratch using NEP+ tools faster and easier than the state-of-the-art robotics framework?

To address RQ1, participants were tasked with installing and using the NEP+ App along with two additional interfaces or modules, each embedding basic robotics functionalities. The first interface captures and publishes images from a webcam to the NEP+ network, while the second interface acquires and publishes human data, including skeleton, hand, and face landmarks, to the NEP+ network. After exploring these interfaces, participants completed the System Usability Scale (SUS), a technology-agnostic questionnaire widely used for assessing perceived usability. SUS comprises 10 items (Table A1), rated on a scale from 1 (strongly agree) to 5 (strongly disagree), resulting in a final score ranging from 0 to 100. Interfaces with a SUS score over 70 are considered acceptable [65]. Table A1 also shows the mean and standard deviation (σ) of participants’ answers for each item. To measure participant feelings toward NEP+ interfaces, we applied a semantic difference (SD) self-reporting questionnaire following recommendations presented by the Kansei Engineering discipline [66]. The SD scale uses two bipolar adjectives with a scale between 1 and 5. While answers closer to 1 reflect positive perceptions towards the proposed interfaces, answers closer to 5 reflect negative ones. All bipolar adjectives and respective results from participant answers are seen in Table A2. We selected a set of bipolar adjectives to evaluate aspects of the model presented in Section 3.3, which are intrinsically subjective and very complex to assess using objective metrics. For example, modern-old and attractive-ugly pairs can be used to assess if the proposed are pleasurable.

Participants found NEP+ interfaces easy to use, straightforward, simple, and clear. They praised the unified and cool design but noted difficulties closing interfaces. Suggestions included a single interface for multiple modules and more information for creating complex systems. Moreover, participants expressed interest in NEP+ interfaces for tasks such as obtaining hand poses and mentioned that their usefulness would depend on potential expansions. They suggested specific robot skills, including data saving, object detection, motion control, human-robot collaboration, motion recognition, and various sensor interfaces for future implementation.

To address RQ2, two activities were designed to assess the workload of developers using NEP+ and ROS2 on non-Linux operating systems. These activities, named activity 1 (involving ROS2) and activity 2 (involving NEP+), each consisted of two tasks. Task 1 required participants to install the respective robotics framework, while in task 2, they needed to create a basic software architecture using the Publisher-Subscriber pattern with the installed framework. Official tutorials for ROS2 and NEP+ were provided to guide participants through these tasks.

Subjective workload measurements were obtained using the NASA Task Load Index (TLX) questionnaire [67], which assesses mental demand, physical demand, temporal demand, overall performance, frustration level, and effort. Part 1 of the TLX questionnaire involves participants rating each dimension on bipolar scales. In part 2, participants make paired comparisons to determine the relative importance of each dimension. The weighted workload score for each dimension is calculated by multiplying the raw workload value from part 1 by the weight value obtained in part 2. Overall workload scores (weighted and unweighted) are classified as very low, low, medium, high, or very high. Mean workload scores for each dimension and overall scores for activities 1 and 2 are presented in Table 3 and Table 4.

Additionally, we registered the time required to complete a task and the number of tasks performed successfully as objective metrics. We give participants at least 5 min to rest and recover from any mental and physical workload between each task of activities 1 and 2. To reduce potential bias, pair participants (P1, P3, P5) were asked to perform activity 1 and then activity 2, and odd participants (P2 and P4) were asked to perform activity 2 and then activity 1. The total time allotted for task 1 was 40 min, and for task 2 was 20 min of each activity. Table 5 and Table 6 show the objective data obtained in activities 1 and 2, respectively. In the case of P3, he decided to use Windows for activity 1 and macOS for activity 2. The other participants used the same computer for performing activities 1 and 2.

## 8. Results and Discussion

This section discusses the results and answers research questions RQ1 and RQ2 according to the data obtained in the experimental evaluations.

### 8.1. Perceived Usability and Feelings about NEP+ Interfaces

The average SUS score obtained from the evaluation was 81, which falls under the rank A usability score category. This result indicates that the overall usability of the NEP+ interfaces is acceptable, as suggested by [68]. The SUS scores obtained for each participant showed that P1 had a SUS score of 82.5, corresponding to category A, while P2 received a score of 65.0, indicating a category of C. P3 achieved a SUS score of 90.0, falling under category A+, and participant P4 received a score of 92.5, resulting in a category of A+. P5’s SUS score was 75.0, placing them in category B+. Table A1 results indicate that participants find NEP+ interfaces easy to use and believe they can be learned quickly. However, some functions, particularly closing interfaces, raised usability concerns. This issue is identified as a usability error and will be addressed in future iterations. Additionally, the semantic analysis results in Table A2 reveal that NEP+ interfaces are perceived as modern, attractive, useful, simple, convenient, and clear.

### 8.2. Can Developers Create Robotic Applications Faster and Easier with NEP+?

Results from objective metrics (time and the number of tasks completed by success), shown in Table 5 and Table 6, suggest that NEP+ tools enable developers to create simple robotic applications faster and more effectively than ROS2 in Windows and macOS systems. More details of this result are described below.

#### 8.2.1. Installing and Using ROS2

In Table 5, two participants failed to install ROS2 on macOS, attributing it to compatibility issues with modern macOS versions. In these cases, the task is considered a failure, the time and NASA-TLX is not registered, and the activity is finished.The remaining three participants using Windows 10 successfully installed ROS2 by following official documentation, but they encountered various bugs, likely related to installation errors when attempting a specific task involving creating and running a Python-based Publisher-Subscriber application. Participant P3, who claimed to have a lot of experience with ROS, considered that "to discover how to fix the bug, it seems like it would take hours" and that "I don’t think that novice users can solve the bug easily". It is relevant to highlight that all participants declared to have several years of experience using ROS. Therefore, results suggest that getting started with ROS2 in non-Linux could be a complex task for most users, including those with experience using ROS in Linux.

#### 8.2.2. Installing and Using NEP+

As shown in Table 6, all participants could install and create a simple Publisher-Subscriber application using NEP+ tools without issues and far behind the time limit. Notably, for participants P1, P2, P3, and P5, it was their first experience using any NEP+ tool or library. Participant P4 was an exception, having previously used the NEP+ library in C# to connect a non-ROS-compatible script with a laptop through ROS over a Wi-Fi network.

### 8.3. Workload Comparison between NEP+ and ROS2

The results from the unweighted workload scores in Table 3 indicate that installing ROS2 in non-Linux environments is associated with high mental demand, time pressure, effort, frustration, and poor performance or satisfaction in task completion. Additionally, using ROS2 results in very high mental demand, time pressure, effort, frustration, and self-performance issues. Conversely, Table 3 suggests that installing NEP+ tools is linked to very low mental demand, effort, frustration, temporal demand, and good performance. However, using NEP+ requires a moderate level of mental demand, temporal demand, and effort since some programming skills are necessary to complete task 2. The findings in Table 3 indicate that the overall workload (both weighted and unweighted) associated with ROS2 in non-Linux environments is high for experienced robotics researchers, while NEP+ results in a lower workload levels.

This pilot test demonstrated that traditional or skilled robotics researchers could benefit from using NEP+ tools. We will use the lesson learned in this pilot test to adapt these evaluations to novice users (such as undergraduate or master students) and non-programmers. For example, while most participants could install NEP+ and ROS2 beyond the time limit, this time must be longer when considering novice users. Moreover, assessment with non-programmers will require using end-user programming tools such as RIZE and tasks with higher abstractions.

## 9. Conclusions and Future Work

We presented an innovative approach to interactive systems development, centering on human aspects and user experiences. The NEP+ framework combines a holistic UI/UX-focused quality model with a software development platform. We illustrated our vision through two real user cases emphasizing collaboration and a human-centered approach. In these user cases, NEP+ tools have to involve individuals from diverse backgrounds in the design of technological advancements.

The model we presented in this article is based on our experience working in multidisciplinary and transdisciplinary teams. However, providing evidence of how this and other similar human-centered initiatives, such as Industry 5.0, have contributed to improving human UX and well-being is a very complex challenge that will require several years to be answered through new collaborations and iterative development. In this direction, this article can be considered as the initial effort.

Results from the pilot test with experienced robotics developers suggested that NEP+ tools enable the creation of software modules faster, easier, and with less mental load and frustration than the state-of-the-art solution in macOS and Windows. Posterior iterations will focus on assessing new versions of NEP+ tools in different contexts and users, such as novice developers and domain experts.

NEP+ is still in its initial development stages. Therefore, it provides few but essential functionalities compared with well-established robotics and IoT middleware. However, NEP+ is designed to empower and coexist with other distributed robotics frameworks using platform-agnostic and high-performance ZeroMQ sockets and interfaces that facilitate their use. Moreover, we proposed a novel vision that can inspire researchers to create robotics modules that novice and expert users can install and use with less effort. Future work will be focused on (i) developing mechanisms enabling the link and easy integration of robotics and IoT middlewares, (ii) proposing workflows and templates for easy development of portable and compatible modules implementing graphical interfaces using Electron and Docker, and (iii) provide solutions able meet the additional user and research needs proposed in the NEP+ design model (i.e., personalization, inclusiveness, data security and use of metrics), (iv) use and evaluate NEP+ middleware features to create Industry 5.0 applications, such as robotic digital twins and Human-Robot Collaboration. Furthermore, we intend to utilize NEP+ interfaces, such as RIZE, to empower and motivate stakeholders and users with different backgrounds to help successfully appropriate or adopt robots in heterogeneous contexts. Updates, interfaces, tools and documentation of NEP+ are available online [41].

## Figures and Tables

**Table 1:** How NEP+ tools proposed in this work can be used to satisfy the quality model proposed in this article.

Quality Aspect	How NEP+ Tools Contribute?
Useful	NEP+ middleware provides development tools enabling users to create low-level modules and execute high-level functionalities (see Section 4)
Maintainable	NEP+ middleware layer facilitates connection and re-use of software modules (see Section 4)
Usable	NEP+ is designed under a iterative interaction design process to ensure usabily of user interfaces and software tools (see Section 7)
Accessible	NEP+ employs web-based frameworks for cross-platform accessibility, enable the use of many programming languages and target diverse type of users (see Section 6)
Convenient	NEP+ simplifies the development of interactive applications by offering user-friendly web-based interfaces, reduced dependencies, and easy installation (see Section 4 and Section 7)
Transparent	This can be achieved by simplifying navigation, maintaining interface simplicity, offering comprehensive documentation, and providing visibility into the system’s operations. In this regard, visualization tools help users understand the current status of the system (see Section 4)
Pleasurable	Web-based frameworks used to build NEP+ tools enable the easy creation of modern, intuitive, and aesthetically pleasing products with a familiar design for general users (see Section 4 and Section 7)
Empower	NEP+ tools has been used to empower users to create interactive applications with physical and digital agents, offering adaptability and inclusivity (see Section 5)

**Table 2:** Integration comparisons related to accessibility aspects between NEP+ and the most popular robotics development frameworks/middlewares. The information displayed in this table was obtained from the official documentation of each framework.

Name	Programming Languages	Operating Systems	Target Users
ROS Noetic Ninjemy	Primarily targets Python 3, Lisp, and C++	Primarily targets Ubuntu 20.04	Robotics researchers
ROS 2 Humble Hawksbill	Primarily targets Python 3 and C++. However, there are community-maintained client libraries for Ada, C, Java, C#, Node.js and Rust	Primarily targets Ubuntu 22.04. However, basic versions have been confirmed to support Windows 10, and macOS 10.14	Robotics researchers, engineers
YARP 3.7.2	Primarily targets C++. However, SWIG bindings for Java, Python, Perl, C#, and Ruby can also be used	Windows (supported version is not specified), and Ubuntu Linux (supported version is not specified), have been confirmed to work on macOS from 10.9	Robotics researchers
NEP+ 0.0.4	Support Python (2 and 3), Java, Node.js, and C#. However, any ZeroMQ compatible programming language (e.g., Rust, Go, Objective-C, Swift) can be supported in the future	Confirmed to work in Windows 10 and 11, Ubuntu Linux 16, 18, 20, and 22, macOS 10, 11, 12 and 13 (including computers using Intel processors and Apple Silicon). NEP Python libraries have been used in Windows 7 and 8	Engineers, novice and non-programmers, practitioners, and robotics researchers

**Table 3:** NASA-TLX results for Activity 1: Installing and using ROS2.

	Task 1: Installing ROS2		Task 2: Using ROS2
**Dimension**	**Weighted**	**Unweighted**	**Weighted**
Mental	256.00	73.00	201.67
Physical	0.00	21.00	0.00
Temporal	206.25	63.00	166.67
Performance	230.00	75.00	287.67
Effort	108.00	49.00	216.67
Frustration	307.00	79.00	350.00
Overall	73.80	59.58	81.44
Interpretation	High	High	Very High

**Table 4:** NASA-TLX results for Activity 2: Installing and using NEP+.

	Task 1: Installing NEP+		Task 2: Using NEP+
**Dimension**	**Weighted**	**Unweighted**	**Weighted**
Mental	67.00	15.00	103.00
Physical	10.00	6.00	0.00
Temporal	45.00	12.00	146.00
Performance	23.00	9.00	47.00
Effort	28.00	9.00	70.00
Frustration	58.75	14.00	44.00
Overall	13.20	10.83	27.33
Interpretation	Low	Low	Low

**Table 5:** Results of task 1 (installing ROS2) and task 2 (using ROS2) of Activity 1. When the task is not completed successfully, time is registered as n/a. The time limit for this activity is 40 min for task 1 and 20 min for task 2.

		Task 1		Task 2	
**Participant**	**Selected OS**	**Time**	**Result**	**Time**	**Result**
P1	macOS	n/a	Failure	n/a	Failure, the participant was not able to complete the previous task
P2	Windows	40 min	Success	10 min	Partial success, completed most steps but encountered an installation error preventing Python script execution.
P3	Windows	35 min	Success	18 min	Partial success, completed several steps but noted issues with ROS2 implementation on Windows.
P4	Windows	38 min	Success	n/a	Failure, basic ROS2 commands did not work, preventing task completion.
P5	macOS	n/a	Failure	n/a	Failure, the participant was not able to complete the previous task.

**Table 6:** Results of task 1 (installing NEP+) and task 2 (use NEP+) of Activity 2. The time limit of this activity is 40 min for task 1 and 20 min for task 2.

		Task 1		Task 2	
**Participant**	**Selected OS**	**Time**	**Result**	**Time**	**Result**
P1	macOS	16 min	Success	13 min	Success
P2	macOS	3 min	Success	10 min	Success
P3	Windows	4 min	Success	3 min	Success
P4	Windows	4 min	Success	4 min	Success
P5	macOS	10 min	Success	12 min	Success

## Data Availability

The data presented in this study are available in insert article.

## Appendix A. Additional Tables

*Usability and Semantic Difference Questionnaires*

**Table A1:** SUS questions and results.

N	Question	Mean	Std Dev (σ)
1	I think that I would like to use these interfaces frequently	4.2	0.40
2	I found these interfaces unnecessarily complex	2.8	1.83
3	I thought these interfaces were easy to use	4.0	0.00
4	I think that I would need the support of a technical person to be able to use these interfaces	1.6	0.80
5	I found the various functions in these interfaces were well-integrated	4.2	0.40
6	I thought there was too much inconsistency in these interfaces	1.4	0.48
7	I would imagine that most people would learn to use these interfaces very quickly	4.6	0.48
8	I found these interfaces very cumbersome to use	1.6	0.80
9	I felt very confident using these interfaces	4.0	0.63
10	I needed to learn a lot of things before I could get going with these interfaces	1.2	0.40

**Table A2:** Semantic analysis results. Feelings/perceptions of participants regarding NEP+ interfaces.

Dimension		Semantic Evaluation	
**Positive (1)**	**Negative (5)**	**Mean**	**Standard Deviation (σ)**
Modern	Old	1.6	0.49
Attractive	Ugly	1.6	0.49
Simple	Complex	1.2	0.40
Clear	Ambiguous	1.4	0.80
Convenient	Problematic	1.8	0.40
Useful	Useless	1.4	0.49
